# Opioid use disorder in pregnancy: leveraging provider perceptions to inform comprehensive treatment

**DOI:** 10.1186/s12913-021-06182-0

**Published:** 2021-03-10

**Authors:** Doris Titus-Glover, Fadia T. Shaya, Christopher Welsh, Danya M. Qato, Savyasachi Shah, Laura E. Gresssler, Rebecca Vivrette

**Affiliations:** 1 Department of Pharmaceutical Health Services Research (PHSR), School of Pharmacy, University of Maryland, Baltimore, USA; 2 Present address: School of Nursing, University of Maryland, Baltimore, Universities at Shady Grove, 9640 Gudelsky Drive, Rockville, MD 20850 USA; 3 Department of Psychiatry, School of Medicine, University of Maryland, Baltimore, USA

**Keywords:** Qualitative pilot study, Medications for opioid use disorder, medication-assisted treatment, Opioid use disorder, Pregnant women, Pregnancy and healthcare provider

## Abstract

**Background:**

Medications for opioid use disorder (MOUD) are recommended with adjuvant behavioral therapies, counseling, and other services for comprehensive treatment of maternal opioid use disorder. Inadequate access to treatment, lack of prescribing providers and complex delivery models are among known barriers to care. Multi-disciplinary provider input can be leveraged to comprehend factors that facilitate or inhibit treatment. The objective of this study is to explore provider perceptions of MOUD and factors critical to comprehensive treatment delivery to improve the care of pregnant women with opioid use disorder.

**Methods:**

A qualitative research approach was used to gather data from individual provider and group semi-structured interviews. Providers (*n* = 12) responded to questions in several domains related to perceptions of MOUD, treatment delivery, access to resources, and challenges/barriers. Data were collected, transcribed, coded (by consensus) and emerging themes were analyzed using grounded theory methodology.

**Results:**

Emerging themes revealed persistent gaps in treatment and challenges in provider, health systems and patient factors. Providers perceived MOUD to be a “lifeline” to women.

**Conclusions:**

Inconsistencies in treatment provision, access and uptake can be improved by leveraging provider perceptions, direct experiences and recommendations for an integrated team-based, patient-centered approach to guide the care of pregnant women with opioid use disorder.

## Background

The use and misuse of opioids nationally has increased in the past decade even among pregnant women [1]. Such use has also led to an increase in opioid use disorder (OUD) [2] and the need for treatment of OUD in this vulnerable population. Yet barriers in access to treatment for OUD [3] remain, and better understanding of the perspective of providers is necessary to craft policies and programs that can effectively improve maternal and child health among women with OUD. The aim of this qualitative pilot project was to augment the literature and explore provider perceptions of OUD treatment and investigate factors critical to comprehensive treatment delivery to improve the care of pregnant women with OUD.

Maternal use of prescription opioids and illicit drugs increased from 1.5 to 6.5 per 1000 deliveries from 2000 to 2014, according to the Centers for Disease Control and Prevention [4]. Under-treatment of pain and provider prescribing practices were contributory factors associated with this increase that has led to a public health crisis [5, 6]. From 2008 to 2012, approximately 39.4% of Medicaid recipients and 27.7% of privately insured reproductive age women (15–44 years), filled a prescription yearly for opioids [7]. In 2014, 21.6% of pregnant Medicaid enrollees filled an opioid prescription [7] and 85.4% of pregnant women in the United States who had cesarean births filled a prescription for opioids (primarily oxycodone) [8].

The increase in prescription opioids has led to an upsurge in use and misuse for a large proportion of women [9]. The incidence of pregnant women with opioid use histories presenting to addiction treatment centers grew from 2 to 28% between 1992 and 2012 [9]. Heroin use grew concomitantly with opioid consumption as an inexpensive derivative. More than half of pregnant women receiving treatment reported a history of heroin use [9].

Opioid use disorder (OUD) [10] is diagnosed by repeated use and occurrences of symptoms such as cravings, tolerance, withdrawal, or difficulty controlling opioid use, or maintaining life events. In addition to physical dependency, OUD predisposes pregnant women to adverse birth and maternal/neonatal outcomes [11]. Death due to unintentional drug overdose has become a major contributor to maternal mortality and a leading cause of pregnancy-associated deaths in states like Maryland and Virginia [12–14]. Concurrently, national incidence rates for neonatal outcomes withdrawal syndrome (NOWS), a collection of symptoms experienced by babies exposed to opioids in utero immediately after birth, have increased by at least five times since 2000 [15–17]. By 2012, approximately 50% of NICU admissions were occupied by newborns with NOWS [17]. Complications for babies with NOWS include prematurity, seizures, feeding and respiratory complications compared to non-opioid exposed infants [15–19].

Comprehensive care management that includes medications for opioid use disorder (MOUD) is recommended for pregnant women with OUD [11, 20–22]. MOUD consists of opioid agonist pharmacological treatment options, such as buprenorphine (Subutex® and Suboxone®) or methadone [20, 22], both proven to be effective in reducing severe withdrawal symptoms, risk-taking behaviors and improving adherence to treatment when combined with behavioral therapies, counseling, and prenatal care [20–22]. MOUD is preferable to medically supervised withdrawal because of the high risk of relapse and adverse outcomes by 54–90% in pregnant women [23].

Methadone therapy induction and maintenance are facilitated through outpatient treatment programs (OTPs) or at inpatient facilities and require daily dosing/visitation [24–26]. Buprenorphine, a partial agonist has unique binding properties which reduces drug-drug interactions and overdose risks [20]. As a result, buprenorphine can be safely started in office-based settings and thus overall, increases treatment availability and decreases stigma [26] compared to methadone dispensing in outpatient treatment facilities [26, 27]. Buprenorphine-exposed neonates reveal less withdrawal symptoms, require 89% less treatment and 43% shorter hospitalization when compared to methadone-exposed babies with feeding and respiratory complications requiring longer hospitalization [28–33].

MOUD provides a critical pathway for recovery during pregnancy, a time when women make changes and invest in behaviors to improve pregnancy and neonatal outcomes [11, 34]. Treatment delivery or implementation, however, is challenging and there is little evidence of strategies that work to improve adherence and maternal/neonatal outcomes [34]. For providers, it is an opportune time to promote best practices and teachings. Among Medicaid recipients only about 50% had access to MOUD for treatment because of reasons such as lack of prescribing providers [35]. Prenatal providers reported complex medical and mental health comorbidities [36] among patients, often outside the scope of any one practitioner, underscoring the need for multidisciplinary coordination. Stigmatization and fear of criminalization of pregnant women by some states impacted treatment delivery [33]. While most studies [27–30] focused on the pharmacological benefits of MOUD, barriers to treatment delivery persist and have not been fully explored among providers.

Given that substantial gaps in treating pregnant women with MOUD remain and mitigating factors have not been fully explored, input from provider stakeholders can influence understanding of effective treatment modalities. Providers who treat, assess and facilitate the care of pregnant women with substance use (in the fields of social work, addiction medicine behavioral medicine, and mental health) have extensive understanding of comprehensive MOUD delivery modalities. Through in-depth qualitative studies researchers can explore underlying issues to guide understanding of OUD treatment in pregnant women [37]. Therefore, the primary objective of this pilot study was to explore factors critical to effective and comprehensive MOUD delivery from the perspective of providers experienced in managing pregnant women with OUD. Specifically, we aimed to understand provider perceptions of facilitators, challenges and barriers to treatment delivery to improve the care of women with OUD during and after pregnancy and inform future research studies.

## Methods

This study was a qualitative descriptive study that used semi-structured interviews from a diverse group of healthcare providers to elicit in-depth data. Health care providers were defined in this study as clinicians and practitioners engaged in treating, assessing, diagnosing, facilitating and coordinating the care of pregnant women. Twelve interviews were conducted by telephone or face-to-face meetings as a commonly used, convenient and efficient method to gain optimal in-depth data and insights into MOUD delivery [38, 39]. Eligible providers included those with experience in maternal healthcare, obstetrics, perinatal mental health, addiction, clinical research, and MOUD. Overall, six individual telephone interviews, one face-to-face interview and two group interviews (2–3 providers/group) were conducted. The popular interview format preference [38, 39] was conducted by telephone, however, one provider who was located in the same building as the study team agreed to a face-to-face interview in an office space. The group format occurred where 2–3 providers were in the same clinical setting, and met during a lunch break to complete the interview in a private and comfortable space. The group format enhanced efficiency and allowed the study to accommodate busy clinician schedules [38, 39]. Each interview lasted approximately 60 min. The study was approved by the Institutional Review Board (IRB) of the University of BLINDED. Verbal permission to audio record was obtained and a facilitator was assigned to recording, time-keeping, and note-taking. Participants were interviewed from October 2017 to August 2018.

**Fig. 1:**
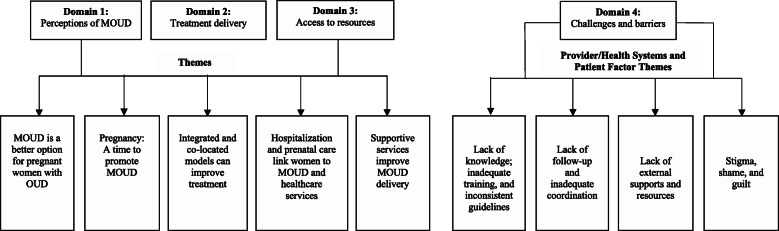
Provider Study Domains and Themes

A gift card incentive was offered to participants who completed the interviews. All interviews were conducted in English. The study was conducted in collaboration with practitioners from the non-academic University of BLINDED and the Women’s Obstetrics Clinic who provided initial referrals. The study team developed 16 open-ended questions based on a report on OUD treatment models of care in primary care settings [34]. Additional questions inquired about practice characteristics and demographics (age, education, years of practice, setting/patient population served). The study questions were also informed by Anderson’s framework for healthcare access [40, 41], reflected in the following four domains (Fig. 1): Perceptions of MOUD; Treatment delivery; Access to resources; and Challenges/barriers. According to Anderson’s framework, provider input and insights are interrelated with the functional needs of patients and communities and are therefore central to understanding access to care and utilization behavior. Interview questions were reviewed for relevance and clarity by two post-doctoral fellows and survey methodologists; and, two subject matter practitioners who modified questions to include practice-relevant probes.

### Recruitment

During the planning phase, the team used convenience sampling methods, including word-of-mouth informal inquiries, to contact clinicians and administrators within the medical network system, who served pregnant women with substance use histories to generate a pool of providers. After compiling a list of names, purposive sampling was then used to recruit providers by email and telephone. Eligibility criteria included providers specialized in obstetrics, perinatal mental health, psychiatry, psychology, behavioral health (child/family), addiction services, and behavioral research, who treat, assess, diagnose, facilitate and coordinate the care of pregnant and postpartum women with OUD. An additional referral source was obtained from providers who had completed interviews. Participants received study-related information/questions to encourage reflection before interviews and to facilitate discussions. Informed consent was received from all participants. Recruitment was conducted by the principal investigator and one post-doctoral researcher receiving training in behavioral health research. The sample size was limited by the number recruited, but we found that the study had also reached saturation at *n* = 12, when provider responses revealed similarities in depth and content.

### Sample

The study sample (*n* = 12) consisted of: one psychiatrist, specialized in addiction medicine; one obstetrician/gynecologist who serves as medical director of an outpatient clinic, one neonatologist/hospitalist; one psychologist in addiction and child/family behavioral health; three social workers responsible for pregnant women and families with substance use histories in outpatient/hospital settings; three midwife/nurse practitioners who worked in both outpatient and inpatient settings; and a mental health nurse practitioner engaged in outpatient clinic settings. Approximately half of the providers were also teaching at the University of BLINDED. The psychologists and social workers had backgrounds in addiction and behavioral health counseling as well as with the child welfare system. Provider professional experiences ranged from 3 to 28 years**.** Five providers (a psychiatrist, three nurse practitioners and the obstetrician/medical director) obtained MOUD training/waivers through SAMHSA, but the only active prescriber during the study was the psychiatrist. Although waivers were recently obtained, it is unclear why the remaining providers were not prescribing MOUD. It is possible that the opportunity for patient induction may not have presented itself, or the referral process to the waivered psychiatrist for prescriptions and behavioral health counseling remained a convenient option for both providers and patients. The psychiatrist had a long history of prescribing MOUD in both outpatient and inpatient settings.

### Analysis

All interviews were transcribed verbatim by a professional agency and analyzed according to an inductive grounded theory approach [42, 43]. The study team leader and one postdoctoral research fellow reviewed the entirety of the transcripts multiple times before beginning coding. Coding was then initiated line-by-line, guided by a starter list generated from interview questions and domains. At least three reviewers from the research team analyzed transcripts and assigned codes for each transcript no less than two times, using Microsoft Word/Excel^©^ (Microsoft Corporation, Redmond, WA, USA) to initiate coding and NVIVO 12 software (QSR International, Victoria, Australia) in the final analysis. Data were finally organized by coding schemes and themes with guidance from an experienced qualitative researcher within the university network who was not part of the research team. A second experienced reviewer outside the university was also consulted about coding and analysis in the final phase. Data were analyzed by domains and organized by process-related broad categories aligned with the inductive nature of qualitative research [37, 38]. The team met three times with expert coders, who did not participate in the interview process, to examine patterns while reflecting on content and referring back to notes and recordings together to check on accuracy and intent. Multiple-tiered processes were conducted to assign and reassign codes with input from the two coders and the team until a consensus was built around overarching themes.

## Results

Emerging themes (Fig. 1) are presented for the four domains: 1. Perceptions of MOUD; 2. Treatment delivery; 3. Access to resources; and, 4. Challenges and Barriers. Provider recommendations are reported separately.

### Perceptions of MOUD

#### Theme 1: MOUD is a better option than no MOUD during pregnancy

Most providers (*n* = 11) perceived MOUD to be a better option for treatment compared to immediate cessation of drug use recommended by some clinicians during pregnancy. Several providers shared the perceptions of MOUD for pregnant women:

“MAT [MOUD] makes so much sense and for most of them [women], it is a lifeline. It is how they survive.

I think without it, they would be back to where they started” (Participant (P) 2, P8).

“Treatment with medications reduces risky behaviors, infections and other negative factors that compromises the health of pregnant women” (P3).

Many providers (*n*=6) argued that the current protocols established for treatment of NOWS were safe and reduced the severity of neonatal withdrawal symptoms. In one provider’s words: “Withdrawal [for babies] outside of the mother is much better than withdrawal inside the mother” (P4).Overall, providers (*n* = 6) with outpatient/inpatient experience found buprenorphine helpful to patients. Two providers shared the following statements:

“Buprenorphine was more likely to lead to successfully being able to stop use. The majority [patients] find it helpful” (P7).

“People in general on buprenorphine feel the effects of the medication less than methadone” (P5).

Most providers (*n*=11) reported that buprenorphine was easily accessible on the streets from prescriptions. A clinic provider reported that about half of the patients receiving buprenorphine knew of the benefits during pregnancy and were “already getting it on the street, to manage their addiction as best as they can” (P5), prior to being started on MAT [MOUD] in the clinic.

#### Theme 2: pregnancy: a time to promote MOUD

Providers (*n* = 10) found pregnancy to be a motivating time for change and stated that:

“Younger women who had not previously sought help for their addiction were motivated to get healthy and found in pregnancy something they want to address” (P5)

One provider spoke about the potential for behavior change during this period:

“Pregnancy offered a precious opportunity to start fresh. A time when patients were the most motivated” (P7).

### Treatment delivery

#### Theme 3: integrated and co-located models can improve treatment

Recent and ongoing discussions about treatment modalities have shed light on the variability of treatment for pregnant women with OUD [34]. Providers operated in practices that were linked to referral services such as medical, addictions and psychiatry specialties, as well as to programs in facilities that were separated but proximal to essential clinics throughout the university networks. Most providers (*n* = 10) were unclear about their practice delivery models, described as “traditional collaborative,” “integrated,” “co-located,” “embedded” or “traditional substance abuse programs.”

Providers (*n* = 10) uniformly described the co-located (or integrated) or “embedded” model as crucial to treatment and viewed the approach more positively than other models. To study providers, practice delivery models were not fully integrated. One outpatient provider commented that the integrated model was “an effective strategy, not 100% co-located care, but as close as we have” (P5). Providers perceived that to be more effective, a co-located model must also be patient-centered.

#### Theme 4: hospitalization and prenatal care link women to MOUD and healthcare services

Hospital admissions provided an opportunity to treat at-risk women who may otherwise be lost to the healthcare system. Providers (*n* = 8) reported an opportunity to “catch them…” (P2) during inpatient admissions when women presented for preterm labor, withdrawal symptoms or other conditions. Two inpatient providers commented:

“We [hospitals] are their entry into getting care. It’s the first time they were getting care for some women” (P1).

“They [women] are referred appropriately and not discharged without an outpatient plan to continue treatment” (P6).

Outpatient clinic providers also described prenatal visits as a “gateway” (P2) for pregnant women with substance use backgrounds to access healthcare.

### Access to resources

#### Theme 5: supportive services improve MOUD delivery

All providers (*n* = 12) acknowledged the critical role of social workers in supporting MOUD delivery initiated by waivered prescribers. One provider spoke about the work of social workers:

“In our clinic, it’s the social worker. We pretty much, the two of us, deal with the bulk of them.

They [social workers] are critical for continuity of care. If we didn’t have a social worker, we couldn't do any of this. We will work with a social worker to get them into an outpatient facility” (P3, P6).

Social workers described engagement opportunities with the healthcare team beyond prenatal appointments such as, screening and assessing new/returning pregnant women, coordinating referrals, locating resources, providing psychosocial support, building relationships and working closely with local/state agencies to identify suitable programs and services.

Regarding postpartum care, all providers (*n* = 12) spoke about the diminishing support women maintained on MOUD received post-pregnancy. Providers described postpartum support as a critical time when women were already overwhelmed with the care of newborns and were therefore at risk of relapsing.

“Women worried about managing their recovery in the context of parenting. Relapse is about two issues: removing the motivator of doing something for their babies… and visitation with providers that occurred weekly in the third trimester…. I worry about the moms once the baby is here and the attention goes away...our ability to help in the postpartum period...is particularly worrisome” (P7).

Another provider concluded:

“You don’t stop being an important person when you are not pregnant anymore” (P1).

### Challenges and barriers

Overall four themes emerged in relation to Provider/Health Systems and Patient Factors, when providers were asked about challenges and barriers to MOUD delivery (Fig. 1).

#### Theme 1: lack of provider knowledge, inadequate training, and inconsistent treatment guidelines

Most providers (*n* = 8) found inpatient medical residents lacked knowledge of current MOUD information and updated guidelines for clinical decision making, often “citing old research” (P9). Inpatient providers (*n* = 4) reported feeling inadequately prepared to screen and assess the increasing number of pregnant women with OUD on admissions and did not “necessarily feel comfortable” (P7, P9) counseling women without adequate MOUD training. Treatment guidelines were reported to be inadequate or absent for post-operative cesarean sections and immediate postpartum. One provider added:

“We don't have written protocols in place. It is an issue when the recommendations can change. Tell us what to do! We want to do the right thing. We want to treat the pain, but not in a way that risks relapse. But then there can be contradictory recommendations given to us” (P7).

#### Theme 2: lack of follow-up and inadequate coordination

For women on MOUD inpatient providers (*n* = 5) complained that there was “no good plan for follow up and coordination” (P5) with prescribing practitioners whose patients were receiving medications. Attempts to communicate with these practitioners during inpatient admissions to determine the right dosage was time-consuming and frustrating when patients did not have prescriber contact information. Outpatient providers (*n* = 5) offered the following comments:

“A lot of them [providers] are very busy, so getting someone to call back and confirm their dose can take too long. A provider would have to seek out notes from other providers to confirm medication dosages” (P7, P12).

A few providers familiar with the justice system (*n* = 3), expressed concerns about MAT [MOUD] access and the criminal justice system. They reported a system that lacked clear guidelines and avenues to communicate with providers and women about MOUD resulting in inadequate or no access to MOUD for incarcerated pregnant women as reported in the following comment by one provider:

“The courts don’t understand when they [women] are on their medication, what the levels are, and might make judgements about the appropriate dose rather than talk to the individual or doctor” (P8).

#### Theme 3: lack of external supports and patient resources

Another theme identified by providers was lack of external supports and patient resources for pregnant women with OUD, potentially a patient-related health systems issue impeding effective treatment**.** Most providers (*n* = 8) stated MOUD delivery was impacted by limited childcare, transportation, and housing. Providers called transportation a major barrier that affected appointments, work, attendance, timeliness, child visitation/child custody and therefore, treatment.

“We don’t have a good way of addressing a lot of other psychosocial needs they have like helping with transportation” (P5).

“I think we forget sometimes how hard it can be to get across town if you don't have a car. If you're trying to bring along a toddler, or you need to go check in with your probation officer or have to provide a document you can't produce…. Every time I hear about some of the things our clients deal with, I'm amazed they're ever there” (P7).

Providers (*n* = 9) complained about lack of access to residential placement facilities and programs for MOUD patients. Few programs existed, particularly for pregnant women with children and those with a critical need such as the homeless or women living with another drug user. Social workers summed up their efforts to locate residential services as beleaguered by:

“Availability, geography, insurance, and transportation... Barriers related to availability of services are the most frustrating... Because when I have a woman who’s willing and able and covered, to not be able to place her in treatment is very frustrating” (P2, P12).

#### Theme 4: stigma, shame, and guilt

In this fourth theme providers revealed stigma, shame, and guilt were considered as barriers to effective MOUD delivery. Providers (*n* = 5) reported women often felt stigmatized by healthcare professionals. Stigma was a barrier to treatment and women were fearful of being discovered to be using MOUD, as stated in the following provider comments:

“For the people I see who are on it [MOUD] to take care of their kid, but they don’t want to have to be on it and they don’t want their children to know they are on it. I have one client whose sisters are on treatment; methadone and she goes over to watch the kids in the car when they go to pick up the medications. They feel very stigmatized of being on it [medication] and needing it” (P3). “For women there is the stigma of being seen in a drug treatment program, which is a major hurdle and discouraged women from keeping appointments. Women were fearful of being discovered to be on MAT” [MOUD] (P1, P11).

“There is also the fear, for example, if they tell other doctors or a pediatrician that they’re on it, that they might indicate they can’t take care of their kids, when they are aware they need to be on it in order to not use. There is a lot of stigma, especially for the people who use it to addiction and pain” (P3).

Providers revealed women reported feeling ashamed and concerned about negative words from healthcare providers and expressed mixed feelings about staying on medications. One provider reported, “I hear nurses all the time say it [MOUD] is replacing one substance for another” (P9) while acknowledging the medication was prescribed for treating patients with OUD. In another area, post-delivery providers (*n* = 6) stated that their patients were stricken by guilt over NOWS. One provider shared this view:

“Part of it is guilt. They don't want to see the baby shaking and going through withdrawal. I think probably the biggest thing that prevents them besides the mechanisms of addiction is the shame and guilt and worry about what's going to happen to their kids” (P8).

## Recommendations

Overall, providers recommended four areas for improving MOUD delivery and focused on both provider barriers/challenges and patient-related factors that impeded treatment progress.

### Improve patient access to resources and education

Most providers (*n* = 10) recommended improvements to psychosocial needs for comprehensive MOUD treatment of pregnant women. Availability and access to patient resources such as transportation, housing, money, support groups, parenting education and outreach workers/recovery support personnel impacted comprehensive treatment: “Lack of safe and stable housing impacts the ability to keep custody of their children, a major stressor for women” (P8). A provider with multiple years working with pregnant women found psychosocial needs to be one of the most important factors for effective treatment and spoke about the importance of available resources:

“Just having more resources, more outreach, some peer recovery navigators and having more access to more residential programs will improve treatment delivery. A peer-to-peer, like another mom who's gone through it and is on the other side, who is in recovery and who was able to keep her kids, I think that would probably be the best help” (P5).

Providers (*n* = 8) wanted access to no-cost/free educational materials from government agencies to reinforce messages to patients about OUD, NOWS and postpartum conditions found to be costly to develop from small budgets. Additionally, child welfare providers recommended anticipatory guidance teaching, “transparency and working with the parents beforehand” (P1) to reduce fears/anxieties, provide clear guidelines/expectations and prevent child removal/custody battles.

#### Advance an integrated “one-stop shop” model

Providers (*n* = 8) recommended an integrated team-based patient-centered approach as the ideal model for pregnant women. Existing evidence-based integrative models appear to be effective. For women with OUD, having related services in one place as a “one-stop shop” facilitates collaboration, coordination and seamless transitions as noted by inpatient providers:

To have a model where the treatment is done within the OB [obstetrics] clinic, would be simple and they might be more likely to follow through [with treatment] if they go to one place. For some women, there’s the stigma of being seen in drug treatment program that bothers them. For some, it’s just convenience or they are familiar with their OB, so they want to stay (P5, P11).

#### Provide education and training to enhance OUD management

All providers recommended education and training to lessen provider discomfort with prescribing medications to pregnant women. Additional provider training will reduce knowledge gaps and can be effective when embedded into a core curriculum in medical education. Providers (*n* = 7) reported that mentorship training with experienced MOUD prescribers such as, psychiatrists will be beneficial to prenatal providers.

“Colleagues would be more willing to obtain the waiver if they had the opportunity to practice. If I were to start in a position where I was expected to prescribe buprenorphine, I would want to spend a little time in the programs where they already do that. Just to make sure I understand all the subtleties” (P7).

#### Reduce stigma and promote patient-centeredness

Providers (*n* = 7) must exercise tolerance towards persons with addictions and understand similarities between addiction and chronic disease management. Substance use disorders may lead to “chronic relapsing just like diabetes and hypertension” (P11).

“No more insulin for you is not an option offered to diabetics after eating sweets and reaching high blood sugar levels” (P11).

Women should therefore not be punished “over a weekend of using” at a time when the need for continued treatment is greatest. Providers should use appropriate terminology and language such as “persons with drug addiction” instead of “a drug addict,” (P6), to reduce stigma and promote patient-centered approaches to advance patient/provider relationships as noted by one provider:

“I think the focus on the relationships is really critical to addressing the roots of addiction, experiences of childhood trauma or addicted parents and patients who did not grow up with a lot of support. In keeping people engaged in recovery and utilizing all the services effectively, I would say relationships are the most important part” (P5, P6).

## Discussion

This pilot study set out to explore provider perceptions and factors critical to delivering comprehensive MOUD (or medication-assisted treatment) [20] to pregnant women. Though medication-assisted treatment remains widely used, the appropriate terminology evolved to MOUD [20, 44]. The overall results revealed several themes related to perceptions of MOUD, provider, health systems and patient challenges/barriers, facilitators and recommendations to guide our understanding of treatment delivery modalities to pregnant and postpartum women with OUD. MOUD is central to comprehensive treatment of OUD in pregnant and postpartum women [11, 20, 44, 45].

Provider insights into the stabilizing effects of MOUD on patients offers insight into strategies that are working to drive future interventions for sustaining treatment. High levels of clinician patient encounters during scheduled pregnancy visits fostered this observation, therefore providers should capitalize on similar opportunities to provide optimum care to women. Healthcare providers have a window of opportunity to promote MOUD recommendations during pregnancy, a time when women are more motivated to change and to utilize health services to a greater extent than at any other time [11]. Therefore, providers can be proactive in assisting women to make early incremental changes between pregnancies to improve overall health outcomes on the journey to recovery. In addition, buprenorphine with its’ 30-day dosing flexibility and convenient prescribing in clinician offices [25, 26] is a beneficial option for women with OUD to regain control of their lives, engage in work-related activities and attend to children compared to methadone that has also shown effectiveness, but has more stringent requirements [27, 29, 30]. The findings of patient self-management, though not widely reported in the literature, offers insights into the lives of women who are often stereotyped and at times criminalized by some states for drug use under “child endangerment” laws during pregnancy [11, 33].

Related provider and health systems themes revealed gaps in knowledge and training, as well as inconsistencies in practice guidelines and outdated information for clinical decision making. Health services themes such as, lack of coordination of services across disciplines and access to qualified prescribing providers remain barriers to comprehensive treatment [11, 33]. Since the study was conducted, however, several initiatives have been implemented to close the gaps in treatment. SAMHSA and other organizations have offered additional online provider education/training on MOUD, and updated clinical practice guidelines to meet the challenges identified [20, 31] A Provider Clinical Support System mentorship [46–48] program has been enhanced and practical hands-on training opportunities have evolved, as recommended in this study. To increase the pool of “qualifying practitioners” to support OUD management, the “*Support for Patients and Communities Act*,” created opportunities for a wider pool of trained practitioners [49] including, “*Clinical Nurse Specialists, Certified Registered Nurse Anesthetists and Certified Nurse Midwives*,” to qualify for waivers. These initiatives can strengthen health systems treatment delivery and reduce barriers to care for women with OUD [46–49].

Waivered providers can be impactful in many settings and to diverse populations, such as incarcerated pregnant women who have no clear path for MOUD continuation [50], as reported in this study. Coordination and follow up [46, 51] across disciplines remains troubling and the extent to which any gains have been made in the care of pregnant and postpartum women since the study was conducted remains unknown at this time. Evaluation studies to determine effective strategies for comprehensive treatment are needed.

Within health systems, diverse treatment practice models such as office-based clinics, OTPs and other treatment access points underscore the complexity of MOUD delivery [20, 34]. As our findings show, it is important to emphasize opportunities for MOUD initiation during inpatient pregnancy-related admissions to improve healthcare access for women who may otherwise delay entry fearing stigmatization and criminalization, including incarceration [11, 50, 51]. Sakala and Corry [52] reported almost all childbirth occur in hospitals; therefore, MOUD can be initiated during admission for untreated patients with OUD, where indicated. Inpatient care is also shaped by multidisciplinary approaches to early identification, referrals and linkages to community agencies to improve care coordination for women with OUD [46]. Increased provider awareness of major access points for early identification has the added value of mitigating potential problems in reproductive age women who are likely to have unintended pregnancies, and who may also find themselves pregnant while using illicit substances [53]. Access to women at risk may be lost if providers fail to recognize the window of opportunity presented during inpatient admissions.

Providers underscored the continuation of outpatient accessibility through clinics [54] in one theme. Traditionally, outpatient prenatal settings were established as major access points for early maternal interventions and services [11]. Given the rise in maternal opioid use, prenatal outpatient settings face multiple challenges to integrate and coordinate addiction services through specialty treatment programs for women with OUD [55]. Effective integrated models often referred to as co-located, characteristically maintain hallmarks of accessibility, coordination and communication [34]. Therefore, maintaining a co-located relationship between outpatient prenatal and specialty addictions services has the potential to improve MOUD delivery.

In another theme, study providers recommended integrated “*one-stop shop*” models, similar to those in comprehensive coordinated treatments for non-pregnant HIV populations that have shown notable success [34, 56]. Implementing such a program, however, will require realigning reimbursements from current sole/primary provider and fee-for-service payer systems (by individual procedure/visit) to value-based bundled maternity or episodic payments for services [57]. The framework for value-based care, quality, coordination of services, interdisciplinary approach and patient-centered care parallels the integrated model proposed in this study. The feasibility of the integrated model needs further evaluation particularly in low waivered provider access areas in rural communities.

Among patient-related health systems factors critical to MOUD delivery, lack of external support such as transportation and residential housing to meet the specific needs of pregnant women (i.e. mothers with small children and pregnant women victimized in domestic violence situations), lack funding support, and remains largely insufficient to address comprehensive treatment [11, 46, 58, 59]. Non-traditional and innovative funding streams can be tapped to assist women [58]. Given the potential for relapse or even death in postpartum [11, 20, 33], supportive postpartum resources can increase treatment adherence and reduce adverse risk outcomes [55]. The role of social workers as the first line of contact highlighted in this study needs further attention. Social workers can extend traditional roles of care coordination across disciplines and multiple settings to improve MOUD delivery to pregnant women. Although Medicaid offers postpartum follow-up through 60 days, researchers and clinicians find the service period inadequate to meet recovery goals [60] and under the Affordable Care Act, not all states have adapted postpartum coverage including addiction and mental health services [61]. Given the implications for health policy, future studies can explore the role of social workers to identify targeted interventions to improve postpartum care and to reduce adverse health outcomes for women with OUD.

Stigma of addiction has been well-documented in the literature [54, 58, 59]. In this study, providers reinforced the call for a cultural shift in thinking about addictions as a chronic disease [62]. A patient-centered approach [63] based on tolerance towards persons with addictions with modifications in the provision of care can decrease stigmatization and improve treatment.

## Strengths and limitations

The study was strengthened by input from an interdisciplinary provider sample within an academic network and health system. Providers freely shared experiences in treating and facilitating the care of pregnant women with MOUD, but the study could have benefitted from the input of additional active prescribing providers. The sample of urban providers may be limited and not generalizable to rural settings.

## Conclusions

Overall, in this pilot study, we presented provider perceptions of MOUD treatment and factors critical to improving treatment in pregnant women with OUD. The study identified emerging themes and insights to guide understanding of treatment, as well as persistent patient, provider and health systems factors that remain challenging. Treatment variances in caring for pregnant women with OUD can be improved by leveraging additional healthcare provider perceptions, direct experiences and recommendations towards a team-based, patient-centered integrated approach. The findings from this pilot study will be utilized to inform larger, future OUD, MOUD and pregnancy studies.

## Data Availability

The data collected and analyzed during the current study are not publicly available due to provider privacy but are available from the corresponding author on reasonable request.

## Abbreviations

ACOG: American Colleges of Obstetricians and Gynecologists
DATA: Drug Abuse Treatment Act
MOUD: Medications for opioid use disorder
MAT: Medication-assisted treatment
NAS: Neonatal abstinence syndrome
OUD: Opioid use disorder
SAMHSA: Substance Abuse and Mental Health Services Administration
SBIRT: Screening, brief interventions, referrals, and treatment
